# Sustainable Bioplastics for Food Packaging Produced from Renewable Natural Sources

**DOI:** 10.3390/polym15183760

**Published:** 2023-09-14

**Authors:** Rajesh Jesudoss Hynes Navasingh, Manoj Kumar Gurunathan, Maria P. Nikolova, Jolanta B. Królczyk

**Affiliations:** 1 Department of Mechanical Engineering, Mepco Schlenk Engineering College, Sivakasi 626005, Tamil Nadu, India; nrajesh@mepcoeng.ac.in; 2 Faculty of Mechanical Engineering, Opole University of Technology, Proszkowska 76, 45-758 Opole, Poland; j.krolczyk@po.edu.pl; 3 Department of Material Science and Technology, University of Ruse “Angel Kanchev”, 8 Studentska Street, 7017 Ruse, Bulgaria; mpnikolova@uni-ruse.bg

**Keywords:** bioplastics, starch, fillers, packaging

## Abstract

It is crucial to find an effective, environmentally acceptable solution, such as bioplastics or biodegradable plastics, to the world’s rising plastics demand and the resulting ecological destruction. This study has focused on the environmentally friendly production of bioplastic samples derived from corn starch, rice starch, and tapioca starch, with various calcium carbonate filler concentrations as binders. Two different plasticizers, glycerol and sorbitol, were employed singly and in a rich blend. To test the differences in the physical and chemical properties (water content, absorption of moisture, water solubility, dissolution rate in alcohol, biodegradation in soil, tensile strength, elastic modulus, and FT-IR) of the produced samples, nine samples from each of the three types of bioplastics were produced using various ratios and blends of the fillers and plasticizers. The produced bioplastic samples have a multitude of features that make them appropriate for a variety of applications. The test results show that the starch-based bioplastics that have been suggested would be a better alternative material to be used in the packaging sectors.

## 1. Introduction

Plastics and other items made of plastic are created from a variety of organic substances that are flexible. Most organic polymers with a high molecular weight and other materials are compounds of plastics (fillers, colors, and additives); usually, they are created synthetically. When referring to unfilled and uncolored plastics rather than compounds, the phrase “natural plastics” is occasionally used in the industry. Every year, 12 million tonnes of plastic end up in the ocean. Of these, 9.5 million tonnes reach the ocean via land, with 1.75 tonnes coming directly from the fishing and shipping industries [1]. It is estimated that there are 51 trillion microscopic fragments of plastic, comprising around 269,000 tonnes. As evidenced by the endurance of natural materials, it is anticipated that since the 1950s, some 1 billion tonnes of plastics have been dumped, some of which may endure for centuries or perhaps substantially longer [2].

Based on how they respond to heat, all plastics may be categorized into the following two basic groups: thermosetting and thermoplastic. Thermoplastics are polymers that can be heated, melted, and molded into the desired shape before cooling. The produced thermoplastic softens and remelts when heated. Polyacrylates, polyesters, polyolefin, polyamides, etc., are examples of well-known thermoplastics. In addition to other products, these polymer materials are used to make packaging, disposable utensils, carpets, lab equipment, apparel, and other items [3]. Unlike thermoplastics, thermosetting polymers are permanently stiffened by the curing of soft solid or liquid resins. Curing is brought on by heat or radiation, and it can be accelerated by adding catalysts. Considerable research has been performed on bioplastics, which are currently the subject of significant research among scientists all over the world due to their susceptibility to water exposure, lack of compatibility, and lower melting point than polymers derived from petroleum [4]. Bioplastics are made from biological or biodegradable components, such as corn starch, food scraps, or even agricultural byproducts. Bio-based plastics are simple to break down in a natural environment, as compared to petroleum-based plastic. They are made from fossil fuels and petrochemical polymers. These results are less negative environmental impacts and global sustainability. The durability of plastics, which is one of their greatest benefits, is also one of their greatest drawbacks as follows: the rate of disintegration (biodegradation) does not correspond to their intended service life, leading to environmental accumulation [5,6].

Compared to commercial plastics used to make polyethylene bags and containers, bioplastics are typically produced at a faster rate [7]. These bioplastics aid in lowering greenhouse gas emissions to reduce environmental pollution [8]. Nevertheless, bioplastics deteriorate gradually depending on the environment’s soil quality [9,10]. Starch, plasticizers, and fillers are the main components of bioplastics in general [11]. Starch-based polysaccharides are thought to be a cost-effective material since they contain a mixture of amylose and amylopectin [12,13,14]. Starch is commonly available and can be found in foods including rice, corn, wheat, potatoes, tapioca, and others; therefore, thermoplastic starch is their primary usage (TPS). Amylopectin and amylose of glucose molecules make up starch, and several types of starches have variable amounts of amylose and amylopectin. Additionally, tensile strength and elongation both increase as the amylose level rises. Plasticized starch will replace synthetic polymers as a material. Tensile strength will rise as molecular interactions and hydrogen bonding intensify. The rigid films’ flexibility will suffer if the tensile strength is too great. Due to package degradation brought on by the environment or product moisture, bioplastic solubility for food packaging applications must be minimal [15,16,17,18]. Mechanical qualities are significant for applications involving food packaging. A sample’s tensile strength varies depending on the type of polymer used, the processing environment, the additives, and the blends. Depending on how it is processed and stored, this will alter. When creating bioplastic samples, several agents, such as additives, catalysts, antioxidants, fillers, and so forth, are added to improve the qualities of the bioplastics [19,20]. According to the tensile and mechanical properties, the main purpose of the fillers is to increase the strength of the bioplastic compound. Starch content is combined to create composite bioplastics, which are formed of the following two major materials: matrix and reinforcement [21,22]. Due to their hydrophilic characteristics, glycerol and sorbitol are used as plasticizers because they have excellent mechanical qualities.

Starches that are compatible with plasticizers, like sorbitol and glycerol, which are inexpensive and abundantly available, are used in the blends of bioplastics. The recyclability of the material is another important factor, and the created bioplastic samples have better mechanical qualities that are comparable to conventional plastics [23,24]. Compared to bioplastics based on individual starch content, the mechanical qualities of composite bioplastics have higher mechanical strength [25,26]. The water solubility and mechanical qualities should be compared to the standard plastic material to replace it for applications such as food packaging [27,28,29]. The composite bioplastics have different starch contents, including rice, corn, and tapioca. The cassava plant, which is readily available, inexpensive, and has qualities like being odorless and colorless, is typically used to extract tapioca starch [30,31]. The amylose and amylopectin content for tapioca starch is 21.2% and 78.8%, respectively. The sample of cassava-based bioplastic is translucent and white in color, and it has a better level of biodegradability. The tensile strength increases with an increase in tapioca starch. The bioplastic sample made of maize starch is transparent. Composite-based bioplastic degrades much more slowly than corn-based bioplastic, and the material’s capacity to degrade is also impacted by humidity. The degradation of composite bioplastics made of cassava and corn starch and cassava-based bioplastics is best at 15% relative humidity. A cassava-based bioplastic degrades substantially more effectively than a corn-based bioplastic when the relative humidity is below 15% [32,33,34]. Understanding the structure and characteristics of the bioplastic compound is made easier by the morphology of the starch content [35,36]. The quick degradation of bioplastic materials will happen as a result of their weak integrity [37]. Above this point, the tensile strength will drop, with an increase in starch content of around 5%, producing an increase. The glycerol level of about 1.5% will be effective up to a point where the plasticizing capability starts to decline. The glycerol will have limited solubility and swell in water if it is kept at 5–10%. Furthermore, strong mechanical properties and resistance were also attained [38,39,40]. The innate nature of protein-based (β-glucans) bioplastics has increased their performance and improved their tensile strength, water vapor permeability, water retainability, and thermal stability under atmospheric conditions. These grains of protein are strongly bonded together through hydrogen bonds that exhibit the enhanced properties of the bioplastics [41,42].

High starch concentrations resulted in a loss of the tensile and mechanical characteristics of albumen/starch-based bioplastic blends. Also, it was observed that the transparency of the film reduced significantly when the starch concentration increased [43]. The bioplastic film made from the potato and rice protein compositions has shown an acceptable range of viscoelastic and water absorption properties that would be utilized in the food packaging industry. The glycerol concentration and thermo molding temperature treatment seem to have an impact on the viscoelastic characteristics of rice protein-based bioplastics. Bioplastics made from potato protein, however, did not appear to be affected [44,45]. As per the researcher, a microbial enzyme, on which use of an aqueous solution with a level exceeding 10%, is required for the bioplastic to prevent microbial growth; therefore, it appears to be the most resilient microbe as a result. Protein-based bioplastics have been studied; however, they are unable to stop formic acid from migrating to water. Gradually moving away from the WG-based matrix, this material is ideal for long-term applications; moreover, essential oil-infused bioplastics may even prevent the growth of germs. These enzymes assist in the creation of an antimicrobial environment inside the container if they are not in direct contact with them [46,47,48]. Recent research has been established on bioplastics to develop the current trend in the bioplastics market. The major contributing factors, such as starch, PLA, and PHA on bioplastic production, provide future implementing ideas onto the market. Bioplastics are favored more in the food packaging industry [49,50]. From research and studies of lateral years on plastics, it is proven that for the next ten years, the bioplastics industry is anticipated to be dominated by non-biodegradable bioplastics, such as bio-based PE, PP, and PET that can be recycled in current systems [51,52].

**Figure 1:**
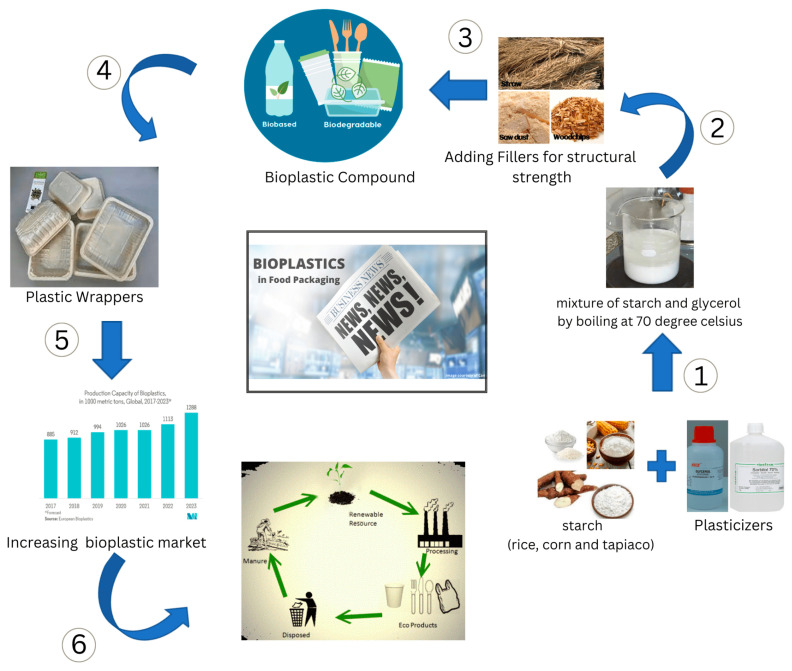
Lifecycle of the bioplastics.

Whey protein bioplastics of biopolymers: natural latex and egg white albumin on combining these and fabricated by compression molding. Water is added as a plasticizer in that mixture. It is found that the addition of about 10% latex and albumin to the whey-based bioplastics would increase its toughness properties and also enhance the characteristics of whey-based materials without compromising their strength and stiffness [27]. This article contemplates that current trends in bioplastics are focused on bio-based technology production rather than conventional methods. Such resource technologies were genetically modified organism cell lines and biomass refinery methods. All these modern bio-based aspects were meant to drive sustainable industry development and regulate the ability of bioplastics to degrade at a certain rate [53,54]. Incorporating glycerol with larger-sized plasticizers, such as xylitol or sorbitol, in the bioplastic film results in the stickiness of the film, promoting separation onto double wall areas and indicating improved tensile strength, stiffness, and oxygen-regulating properties. Thermoplastic starch-blown films having high quantity of plasticizers would not be recommended due to their high water/moisture sensitivity and surface stickiness [55]. In biochemical and soil conditions, PLA breaks down quickly for about a few weeks [56]; however, because of its high price and excessive brittleness relative to typical synthetic materials, it is not extensively utilized. Plastics that have poor mechanical characteristics are typical of PLA composites made with other natural polymers. The natural polymer and the PLA matrix were not bound well together. Recently, polyethylene glycol, polyethylene, glucose, monoesters, and partial fatty acid esters have been utilized to enhance the flexibility and impact resistance of PLA. Many compounds, including citrate esters, have been tested as plasticizers. As a result, PLA polymers’ properties and possible uses have been identified and they are greatly improved [57,58]. The starch is promoting a pathway for the manufacturing of bioplastics, which could result in the creation of materials with exponentially better performance in the food packaging industries. It follows that the properties of various materials are connected to how well starch materials cling to them and how they are compounded. As thermoplastics are processed using extrusion technology, which is one of the basic techniques that has been investigated and developed to treat starch-rich products [59]. The solubility of the substance and the values of the intrinsic viscosity of the synthetic component both demonstrate the remarkable transformation of the structure of the unstabilized sample during photo-oxidation. The positive effect of the stabilizers on the durability of produced biodegradable polymer would have been interpreted by the amount of absorbance proportionate to a lower wavelength region of these compounds [60]. Biopolymers are polymers derived from renewable biological sources, such as plants, animals, and microorganisms. They offer several advantages over traditional petroleum-based polymers (plastics) and have gained increasing interest in various applications due to their eco-friendly and sustainable nature. Biodegradability, compostability, energy-efficient processing, reduced dependence on fossil fuels, non-toxic, and safety are some of the key advantages of using biopolymers. While biopolymers offer many advantages, it is important to note that their adoption is not without challenges. Issues such as cost, scalability, performance, and competition with well-established petroleum-based polymers remain considerations for widespread implementation. Nonetheless, ongoing research and technological advancements continue to address these challenges and further expand the use of biopolymers in various industries. Figure 1 illustrates the lifecycle of the bioplastics.

This investigation focuses on the use of renewable waste from organic agricultural sources, such as corn starch, rice starch, and tapioca starch, to make bioplastics. Using widely available, plentiful, biodegradable, and renewable natural waste as reinforcing fillers, can help reduce the risks and problems associated with conventional plastics as well as the degradation of mechanical properties.

## 2. Materials and Their Properties

### 2.1. Starch

The two polysaccharides amylose and amylopectin make up the granular structure of starch, which is very cheap and abundant in nature. These starch-based materials are biodegradable and thermoplastic.

#### 2.1.1. Corn Starch

**Figure 2:**
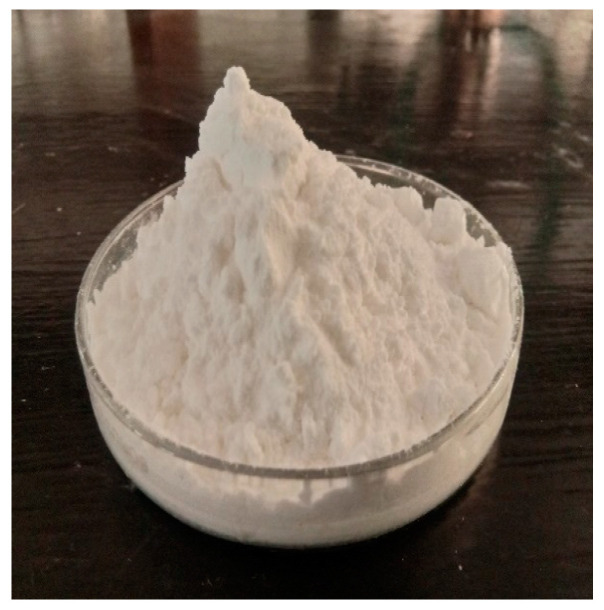
Corn starch.

One of the cereal starches obtained from maize is corn starch, which is mentioned in Figure 2. Commonly recognized for its white color and wide range of uses, including the food sector and adhesive preparations; moreover, it serves as a component in cosmetics. These starches are made up of a 1:3 ratio of two distinct polymers, amylose, and amylopectin.

#### 2.1.2. Rice Starch

**Figure 3:**
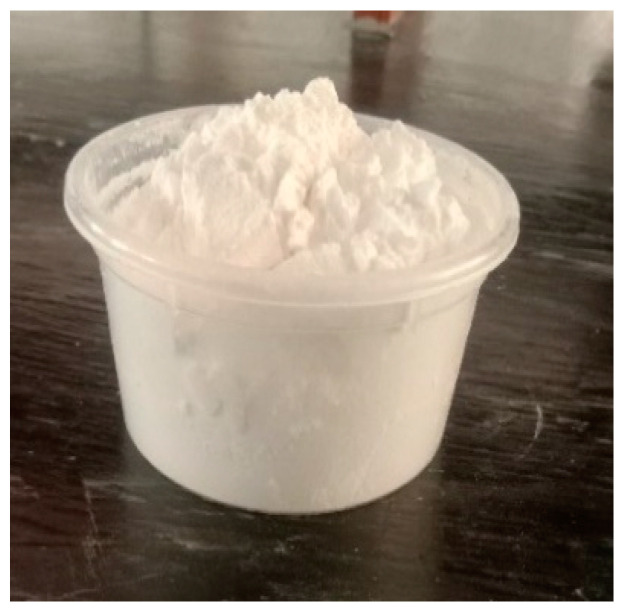
Rice starch.

The milled rice has a significant amount of rice starch. Using the dry techniques, this rice starch comprises glucans, as shown in Figure 3. These starches are known for their color and gel-like properties. As with any bioplastic, the environmental benefits of using rice starch in bioplastic production are closely tied to proper disposal practices.

#### 2.1.3. Tapioca Starch

**Figure 4:**
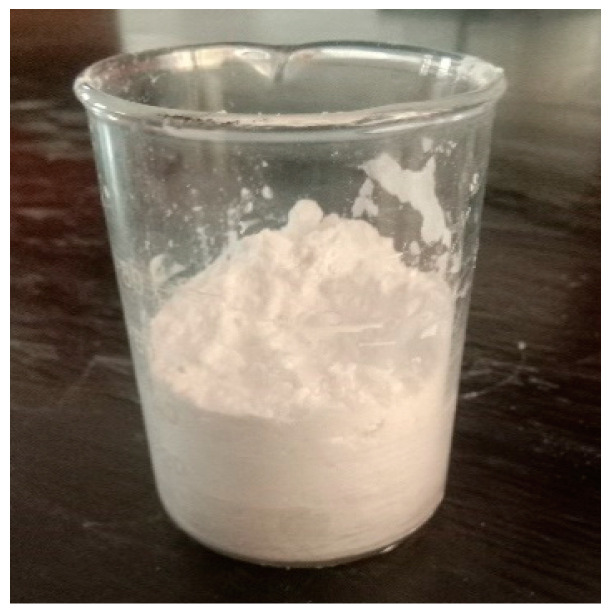
Tapioca starch.

The cassava plant is used to obtain tapioca starch, a relatively accessible substance. These starches typically have hydrophilic properties. These starches are indigenous to Brazil’s northeast, as depicted in Figure 4. The thickening agent for food applications is frequently tapioca starch. Amylopectin (85%) and amylose (15%) are both present in this tapioca starch.

### 2.2. Plasticizers

Plasticizers are essential additives used in bioplastics and conventional plastics to enhance their flexibility, durability, and processability. They are incorporated into plastic formulations to improve the material’s performance and make it more suitable for various applications.

#### 2.2.1. Glycerol

**Figure 5:**
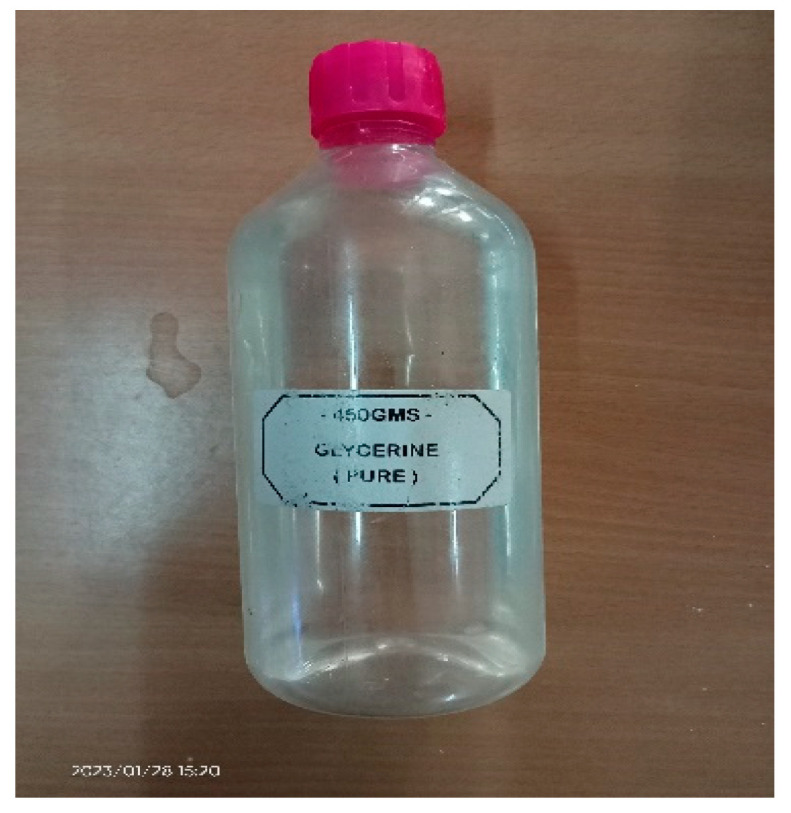
Glycerol.

Glycerol is a viscous liquid that is odorless, non-toxic, and used in numerous applications as a solvent and lubricant according to Figure 5. A stable hydrophilic biopolymer chain is provided by glycerol.

#### 2.2.2. Sorbitol

**Figure 6:**
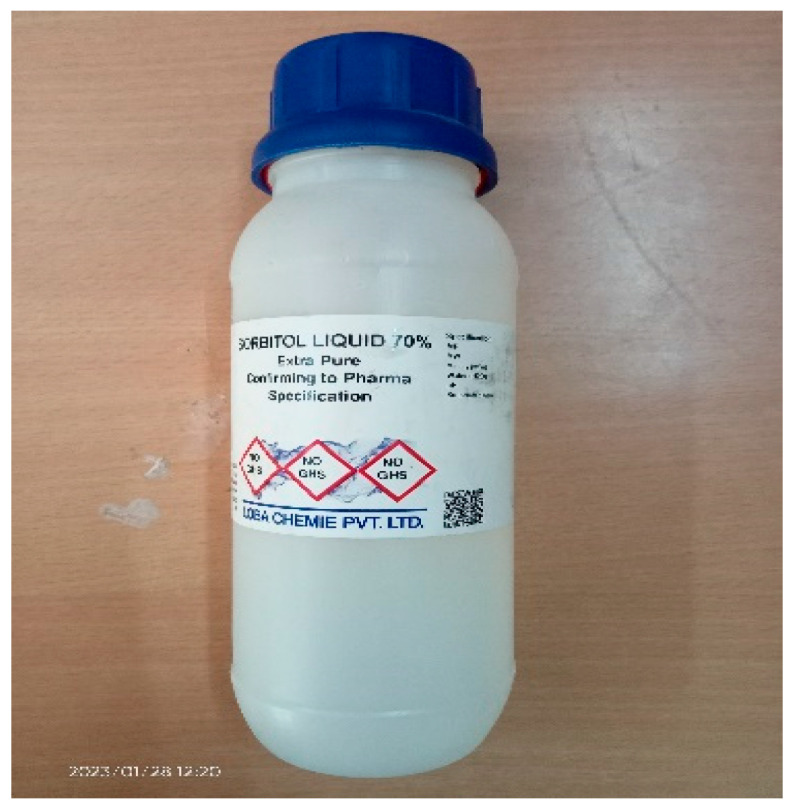
Sorbitol.

Sugar alcohol is the common name for sorbitol. They are produced when glucose is reduced. In numerous applications, this substance is frequently utilized as a sweetener and texturizing agent as shown in Figure 6. It serves as a renewable feedstock, helping to create a more sustainable and eco-friendlier bioplastic alternative to conventional plastics.

### 2.3. Fillers

Fillers are additives used in bioplastics to improve certain properties or reduce production costs. These fillers are typically inert, non-toxic materials that are mixed with the biopolymer matrix to achieve specific performance enhancements.

Calcium Carbonate

**Figure 7:**
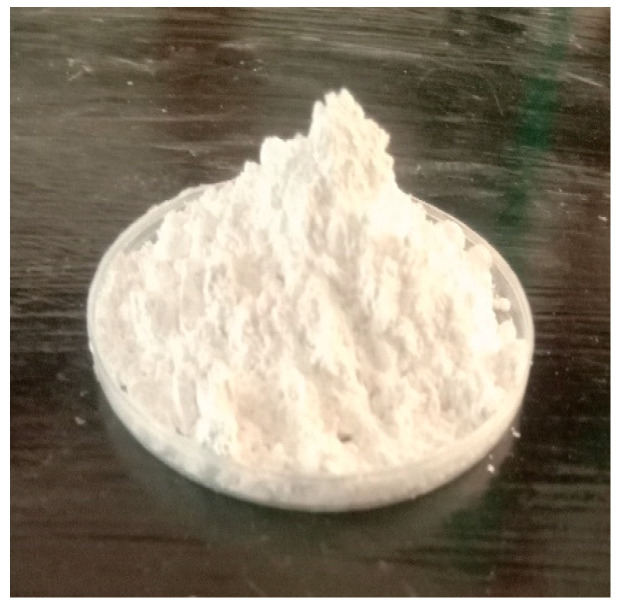
Calcium carbonate.

Calcium carbonate (CaCO_3_) is frequently used as filler to lower costs and enhance the materials’ mechanical qualities. Calcium carbonates (Figure 7) are employed in many different applications, such as the production of polyesters, in addition to thermoplastics.

## 3. Methodology

The bioplastic sample preparation was performed using a variety of techniques. The best practices for efficient binding of the sample on the process compound materials would be accomplished by the following: weighing out materials at suitable standard levels, such as the starch content of the corn, rice, and tapioca that are essentially required for making the sample. The first stage in the procedure is to calculate the appropriate ratio of each starch, such as rice, corn, and tapioca. The measured starch powder is then added to the beaker, followed by the preparation of the plasticizers, 5 mL of glycerol, and sorbitol, which are required as important plasticizers for sample production. Then, 95 mL of distilled water is added to a beaker. They are combined by using a glass rod. The weighted starch content beaker is then filled with the plasticizer mixture, which is then quickly mixed with it using the same glass rod to produce a turbid white solution. The magnetic hot plate device that had been used to heat the solution is turned on. Meanwhile, the glass rod is used to stir the solution continuously to ensure full coagulation of the solution. After reaching a paste-like state, a basic glass plate with an aluminum foil covering is made. The paste mixture is then distributed across the whole glass sheet plate, giving a sample a definite sheet structure when it is allowed to cool for three to four consecutive days. Different compositions of materials are presented in Table 1.

### 3.1. Preparation of Rice Starch

To obtain rice starch in powder form, 1 kg of paddy rice is placed in a container with enough water to cover it, and the rice is allowed to soak for around 2–3 h. Then the mixture of rice and water is blended in a mixer grinder to obtain a semi-liquid form. To separate the water content and husk from this combination, by using a fine, lint-free white cloth. The dissolved starch is present in the water; thus, when the water content is dried off, the starch will be in powder form.

### 3.2. Characterization Methods

#### 3.2.1. Solubility in Water

**Figure 8:**
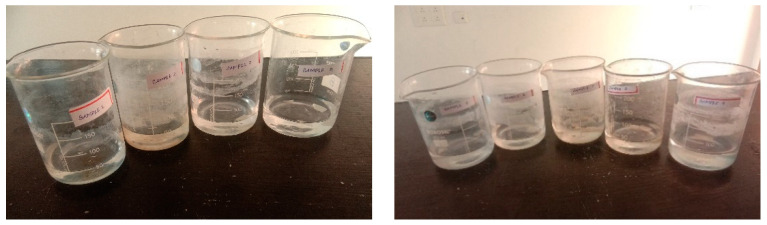
Solubility in water.

In total, nine bioplastic sample pieces totaling 1.5 cm^2^ were cut. It is weighed and measured to determine the sample’s starting weight (W_1_). The sample is then submerged in 50 mL of distilled water and left there for 24 h at room temperature, as shown in Figure 8. The samples were removed from the water by filtering it after 24 h. The remaining samples that had been filtered were dried in an oven at 85 °C for 24 h before being weighed as the final product (W_2_). The following formula was used to determine the solubility in water:

(1) $$\mathrm{Solubility}\;\mathrm{in}\;\mathrm{water}\;(\%)=\frac{W_{1}-W_{2}}{W_{1}}\times 100$$

W_1_—weight of the initial sample in g, W_2_—weight of the dried sample in g.

#### 3.2.2. Solubility in Alcohol

**Figure 9:**
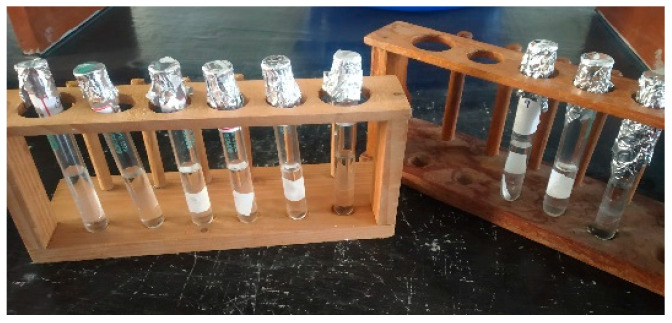
Solubility in alcohol.

Nine bioplastic sample pieces totaling 1.5 cm^2^ were cut. It is weighed and measured to determine the sample’s starting weight (W_1_). It is then submerged for 24 h at room temperature in 3 mL of ethanol in a 10 mL test tube that is sealed, as depicted in Figure 9. The samples were extracted from the ethanol after 24 h by filtering it. The remaining samples that had been filtered were dried in an oven at 85 °C for 24 h before being weighed as the final product (W_2_). The following formula was used to determine the solubility in alcohol:

(2) $$\mathrm{Solubility}\;\mathrm{in}\;\mathrm{alcohol}\;(\%)=\frac{\left(W_{1}-W_{2}\right)}{W_{1}}\times 100$$

W_1_—weight of the initial sample in g, W_2_—weight of the dried sample in g.

#### 3.2.3. Moisture Content

**Figure 10:**
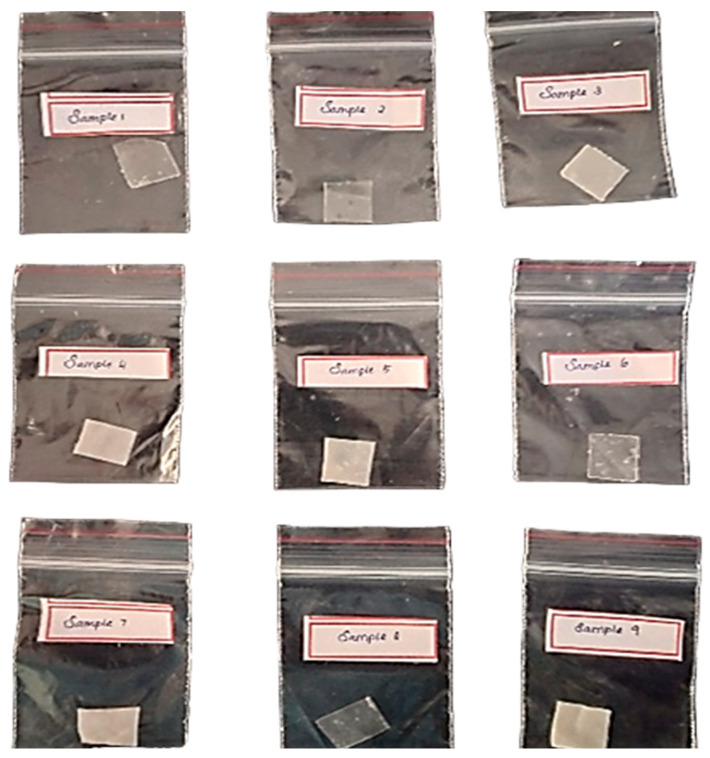
Bioplastic samples.

Nine bioplastic sample pieces totaling 1.5 cm^2^ were cut. It is weighed and measured to determine the sample’s starting weight (W_1_). The samples were prepared in an oven at 85 °C for 24 h, according to Figure 10, and the final weight of the samples was calculated (W_2_). The following formula was used to determine the moisture content:

(3) $$\mathrm{Moisture}\;\mathrm{content}\;(\%)=\frac{\left(W_{1}-W_{2}\right)}{W_{1}}\times 100$$

W_1_—weight of the initial sample in g, W_2_—weight of the dried sample in g.

#### 3.2.4. Absorption of Water

All the samples of 1.5 cm^2^ bioplastics were dried in an oven at 85 °C for 24 h. The dried samples were then weighed and measured (W_1_). The dried samples were then submerged for 24 h at room temperature in 50 mL of distilled water. The weight of wet samples was weighed and measured after 24 h (W_2_). The following formula was used to determine water absorption:

(4) $$\mathrm{Absorption}\;\mathrm{of}\;\mathrm{water}\;(\%)=\frac{\left(W_{2}-W_{1}\right)}{W_{1}}\times 100$$

W_1_—weight of the initial dried sample in g, W_2_—weight of the final wet sample in g.

#### 3.2.5. Biodegradability Test

Nine samples of 1.5 cm^2^ bioplastics were weighed, and the initial weight was noted (W_1_). The styrofoam cups are used to collect the garden soil. The samples were stored at room temperature for 5 days after being buried in 2 cm of damp soil. For five days, the soil needs to be kept moist. The samples were taken after five days and dried for 24 h in an oven set to 85 °C [11]. The dried samples were weighed and recorded as final weight after 24 h (W_2_). The following formula was used to determine the biodegradability:

(5) $$\mathrm{Biodegradability}\;(\%)=\frac{\left(W_{1}-W_{2}\right)}{W1}\times 100$$

W_1_—weight of the initial sample in g, W_2_—weight of the dried sample in g.

#### 3.2.6. Tensile Strength

Using the universal testing machine (UTM), samples of bioplastics were tested for tensile strength according to ASTM D638-03 standards [61]. The samples were divided into cross sections that measured 60 mm in length by 15 mm in breadth, with a 40 mm gap between each strip. The machine runs at a crosshead speed of 10 mm/min. Each sample’s tensile strength was noted, and all the samples were tested.

#### 3.2.7. Scanning Electron Microscope (SEM) Analysis

Using a scanning electron microscope (HITACHI-Japan made, Tokyo, Japan), the morphological structure of the bioplastics samples was examined. Samples of 1.5 cm^2^ in size were used, and a 62 µm emission current was used to power the gadget. For various magnifications, the accelerating voltage of 10 kV was applied at a working distance of 9 mm. The gold was used to layer the samples before the analysis of SEM.

#### 3.2.8. Fourier Transform Infrared Spectroscopy (FTIR)

All samples of bioplastics were subjected to FTIR analysis using the FTIR-8400S equipment (Shimadzu, Kyoto, Japan) with 24 scans, 1 cm^−1^ resolution, and wavenumbers between 4000 cm^−1^ and 500 cm^−1^. The peaks are obtained for the corresponding wavenumbers. The bonding type of molecules like O-H, C-H, and other compounds was determined by the peaks. The spectrum in graphical form was obtained and analyzed in the results. The graphical spectrum was obtained and examined in the outcomes.

#### 3.2.9. Thermal Analysis

In the instrument TGA 5500, thermogravimetric analysis (TGA) was carried out. The temperature was raised from room temperature to 400 °C, while increasing by 15 °C/min for the bioplastic samples, which ranged in weight from 2.6 to 8.3 mg.

### 3.3. Statistical Analysis

The Grey Relational Analysis of the Taguchi method is used for the optimization procedure. Here, components such as starch, glycerol, sorbitol, and fillers are considered. The results from tests for solubility in water and alcohol, water absorption, moisture content, biodegradability, and tensile strength are used to determine the outcomes. Here, the Taguchi analysis makes use of a three-level design and L9 runs. Finding the normalizing sequence of experimental data with a range of 0–1 is the first stage in the Grey Relational Analysis. The values of solubility in water and alcohol, water absorption test, and moisture content in this process all adhere to the principle of “smaller is better,” which is expressed as follows:

(6) xi*=max xi⁡k−xikmax⁡xik−minxik

The values of biodegradability and tensile strength adhere to the principle of “bigger is better”, which is expressed as follows:

(7) $$x_{i}*=\frac{x_{i}\left(k\right)-minx_{i}\left(k\right)}{\max x_{i}\left(k\right)-minx_{i}\left(k\right)}$$

where x_i_* (*k*) is the value following Grey Relational Generation (*i* = 1, 2, 3,…9), min *x_i_*(*k*) is the least value of *x_i_*(*k*), max *x_i_*(*k*) is the greatest value of *x_i_*(*k*), and *k* = 1, 2, 3 for the kth results. Table 2 displays the results normalization sequence and Table 3 presents the deviation sequence.

Finding the deviation sequence is necessary after calculating the normalizing sequence. It is the difference and the reference sequence ${(x}_{0}^{*}\left(k\right))$ compatibility sequence $\left(x_{i}k\right))$, and which can be expressed as follows:

(8) $$\Delta_{oi}=x_{0}^{*}\left(k\right)-x_{i}\left(k\right)$$

where $x_{0}^{*}\left(k\right)=1.000$.

To illustrate the link between the ideal and real normalized experimental results, the Grey Relational Coefficient (GRC), which is derived as follows:

(9) $$\xi_{i}(k)=\frac{\Delta_{min}+\psi \Delta_{max}}{\Delta_{oi}\left(k\right)+\psi \Delta_{max}}$$

ψ—distinguishing coefficient, which lies between 0 and 1.

Here, take as $\psi =0.5.\;\Delta_{min}$—minimum value of deviation sequence and $\Delta_{max}$—maximum value of deviation sequence.

Table 4 shows the Grey Relational Coefficient. After finding the Grey Relational Coefficient, the Grey Relational Grade is calculated by averaging the Grey Relational Coefficient.

A greater GRG value indicates that the matching sequence is nearer to the optimal according to the GRG results. Sample 1 has a higher GRG value, per the GRG results; thus, it might be selected as optimal.

The Taguchi analysis in Minitab software (version 19) is then run after feeding the causes and answers. The response table for GRG is likewise prepared after calculating the mean of GRG for the other parameters. The rank and delta values are also provided. The ideal graphs for GRG are produced between the factors and mean values. According to the response table and graph, the highest-grade values for each factor are chosen, making starch (Level 1), glycerol (Level 3), sorbitol (Level 1), and fillers (Level 1) the best choices. The final product is made up of a combination of 20 g of starch, 5 mL of glycerol, 0 mL of sorbitol, and 0 g of filler. Table 5 shows the response for Grey Relational Grade. Table 6 shows the Grey Relational Grade.

## 4. Results and Discussion

### 4.1. Solubility in Water

**Figure 11:**
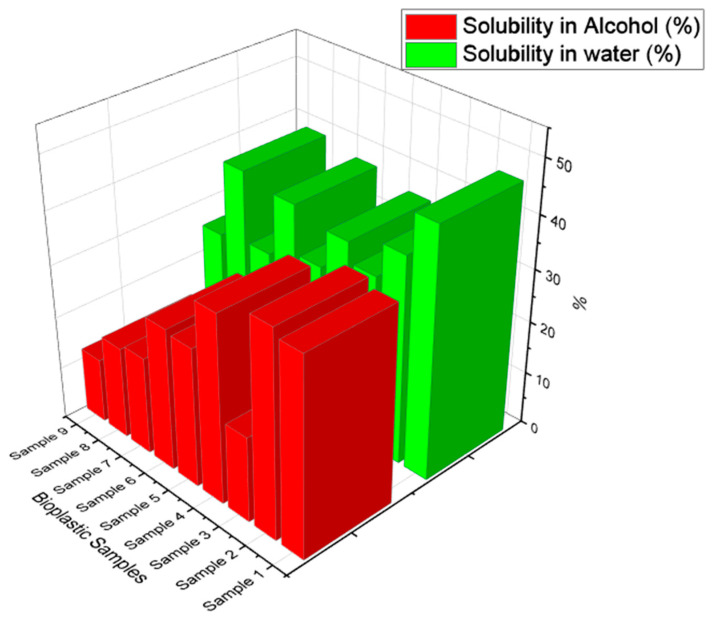
Water and alcohol solubility on bioplastic samples.

The interaction between the molecules of starch and the plasticizers, such as glycerol and sorbitol, with the addition of water can be used to explain this technique of experiment. The solubility in the water experiment yields results that improve the material’s intended water resistance. Figure 11 can be used to explain these experiments. When compared to other samples, like Sample 5, Sample 9, and Sample 2, Sample 1 has the widest range of solubility in water. According to this, the sample made with the inclusion of glycerol as a plasticizer has a higher solubility in water than the sample made with sorbitol as the plasticizer (Sample 5).

These findings also show that Sample 7, a sample made using calcium carbonate as filler, has a lower water solubility than the other samples. According to these findings, glycerol is more attracted to the hydrogen bond, and because they have a lower molecular weight than sorbitol, this occurs. The protein composition based on the starch used to prepare the samples affects the solubility in water. These experimental findings indicate that the sample’s solubility in water depends on the interactions between the amylose and amylopectin contents of the starch. Previous studies have indicated that a bioplastic’s solubility in water is influenced by the type of plasticizer [62]. The properties of these sample findings vary depending on the amount of sorbitol, calcium carbonate, and glycerol added as fillers.

### 4.2. Solubility in Alcohol

These results are similar to the features of solubility in water and follow the results of solubility in water. According to the graph shown in Figure 11, samples made with glycerol as a plasticizer had a higher solubility in alcohol than samples made with sorbitol alone or in combination with both plasticizers. Sample 2 has a higher solubility in the alcohol, which is composed of plasticizers such as glycerol. Samples 8 and 9 had reduced solubility in alcohol since they were made by mixing glycerol and sorbitol. The solubility of a substance is mostly determined by the disappearance of glycerol or sorbitol, which is only marginally soluble in alcohol in comparison to water due to the starch content, which is insoluble in alcohol at ambient temperature.

The samples created by adding calcium carbonate as a filler produce different outcomes. The solubility level of samples of bioplastic is reduced by the addition of fillers like calcium carbonate. This may be demonstrated using Samples 3, 7, and 9, which were created by adding fillers such as calcium carbonate. The inclusion of fillers typically causes the material’s porosity, which prevents it from being soluble in alcohol according to the results of earlier studies [63].

### 4.3. Moisture Content

**Figure 12:**
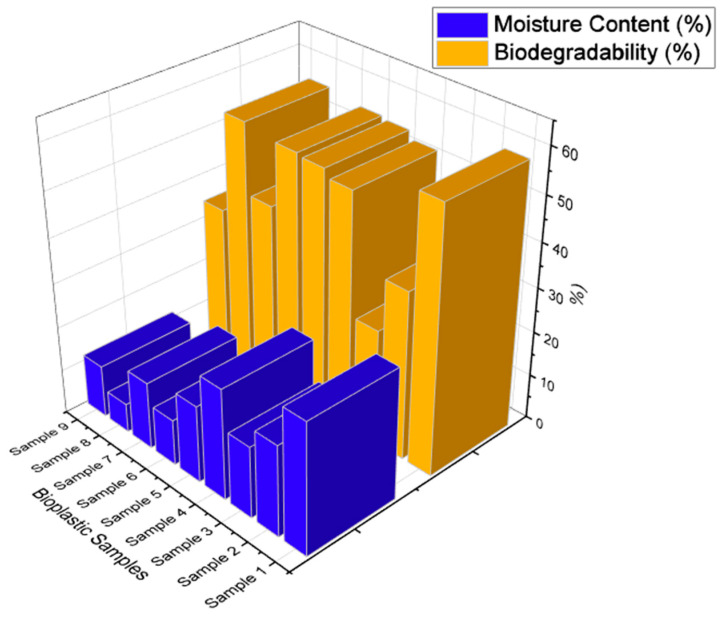
Moisture content and biodegradability on bioplastic samples.

The values of the moisture content are shown in Figure 12 and note the moisture content of the prepared bioplastic samples. It was found that samples made from glycerol have a high moisture level, with Sample 1 having a moisture value of 29.63%. When compared to bioplastic samples made from glycerol, the sorbitol sample has a moisture content that is less than 20%, which is noteworthy. This is because the presence of the starch molecules’ long-lasting hydrogen bonds with them will result in a lesser attraction for water molecules.

Glycerol, on the other hand, contains a hydroxyl group that promotes the formation of hydrogen bonds and increases the attraction of water molecules. Sample 8, which was created by combining sorbitol and glycerol to create bioplastic samples, had the lowest moisture content measurement, at 6.5%; moreover, the moisture content value increased slightly if the filler, i.e., calcium carbonate (CaCO_3_), was added to the sorbitol. It should be noted that adding a filler caused the moisture content to rise by 5%. A drop in moisture content value was seen if the filler was also added to the glycerol at the same time. The moisture content value increased by 5% when the filler was added to the mixture of glycerol and sorbitol. Such outcomes were seen in earlier research studies as well [64].

### 4.4. Biodegradability Test

The physiochemical characteristics of a bioplastic sample, such as its chemical structure, affinity for water, molecular weight, and others, determine its biodegradability. Without the use of fillers, it has been observed that biodegradation will rise. There will be a decrease in the percentage of biodegradability when the fillers are added. It has been discovered that the mixture of sorbitol and glycerol-based samples (Sample 8) has a high capacity for biodegradation due to the samples’ affinity for water. Half of the samples’ capacity to degrade when combined with these plasticizers is reduced when fillers are added (Sample 9), as depicted in Figure 12. Both biodegradation and oxidation are supported by natural fillers. Following a previous study, the amount of filler increased the biodegradation (weight loss percentage) of biocomposites, and that increase was proportional to the filler’s amount [65].

But when samples are made using sorbitol, fillers do not impact how quickly they degrade. An increased filler weight will result in a modest improvement in biodegradability, which also applies to Sample 4. Sample 3 has an extremely poor biodegradation capacity, with a 25.73% score. It can be the result of the sparing use of fillers. These findings support the notion that prepared samples have higher biodegradability.

### 4.5. Absorption of Water

**Figure 13:**
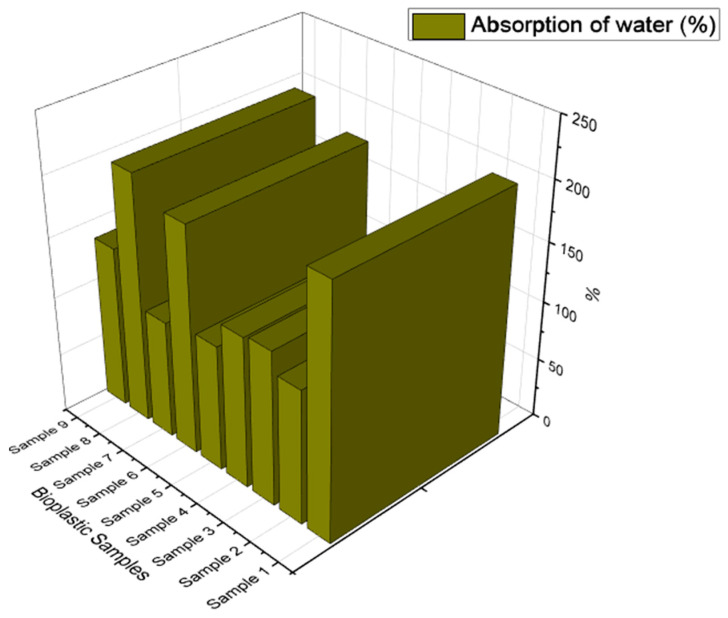
Absorption of water on bioplastic samples.

Prepared bioplastic samples have a significant absorption value that has an impact on the probability and of the samples. Figure 13, for example, in Sample 1, the values show the highest range despite involving glycerol as a main plasticizer, which induces the composite starch particles that are closely bound together. Observing the composite starch, the corn starch (1/3rd of total weight) has an intensive rate of retentivity towards the glycerol because of the presence of amylose (a straight chain glucose molecule) and amylopectin (a compounding polysaccharide structure, which enables correlation and continues chain bonding of glucose molecules) these two polymers are responsible where these glucose molecules are broken down it enables easy permeation of water. Similar findings were made in the earlier work when it was shown that adding sorghum stalk filler to a bioplastic made of sorghum starch increased the amount of water that was absorbed [66].

Because sorbitol is a significant plasticizer, the coagulation and binding of the components occur well, and the contents exhibit less porosity, eliminating the inclusion of pores In Sample 7 the absorption value is the lowest of all of them. When both plasticizers are added, the amount of calcium carbonate acts as a filler, which reduces the rate of absorptivity. Samples 8 and 9 shows high and low values despite the contexts of starch proportions being the same, whereas the filler on Sample 9, in a small amount, would characterize the least value.

### 4.6. Tensile Strength

**Figure 14:**
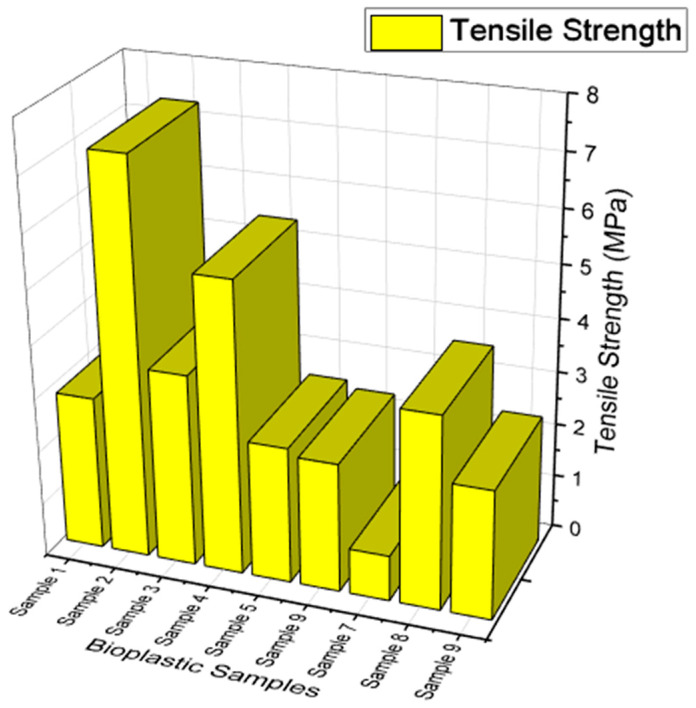
Tensile Strength on bioplastic samples.

For use in food packaging, it was crucial to examine the tensile strength of the bioplastic samples. The tensile strength values for the bioplastic samples are shown in Figure 14. The tensile strength of the produced bioplastic samples was determined using the universal testing machine. It was found that the plasticizers affect the manufactured bioplastic samples’ tensile strength. When compared to a sample made from sorbitol without the inclusion of fillers, the bioplastic samples made from glycerol have a lower tensile strength. The bioplastic sample made from glycerol is found to have a maximum tensile strength of 7.38 MPa (Sample 2). When molecular interactions and hydrogen bonding develop, the maximum tensile strength of the bioplastic sample made from sorbitol is found to be 13.612 MPa (Sample 6). It was found that adding fillers improved the tensile strength of the bioplastic samples proportionately. Similar findings were obtained from an earlier study on the tensile strength of bioplastics made from starch [67].

The inclusion of fillers, such as calcium carbonate (CaCO_3_), has an impact on the bioplastic sample’s tensile strength as well. It was discovered that the glycerol’s tensile strength improved when CaCO_3_ was added. The sorbitol’s tensile strength was decreased when CaCO_3_ was introduced. The sorbitol with calcium carbonate (CaCO_3_) sample was determined to have the lowest tensile strength value, measuring 0.85 MPa (Sample 7). Both plasticizers will affect the tensile strength of a sample when they are mixed. Better tensile strength was produced. The addition of calcium carbonate was found to have decreased the tensile strength (CaCO_3_). Also, it was found that when the starch level rose, the samples’ ability to withstand tensile stress decreased. The samples made from glycerol were also substantially more flexible than those made from sorbitol.

### 4.7. Scanning Electron Microscope (SEM)

**Figure 15:**
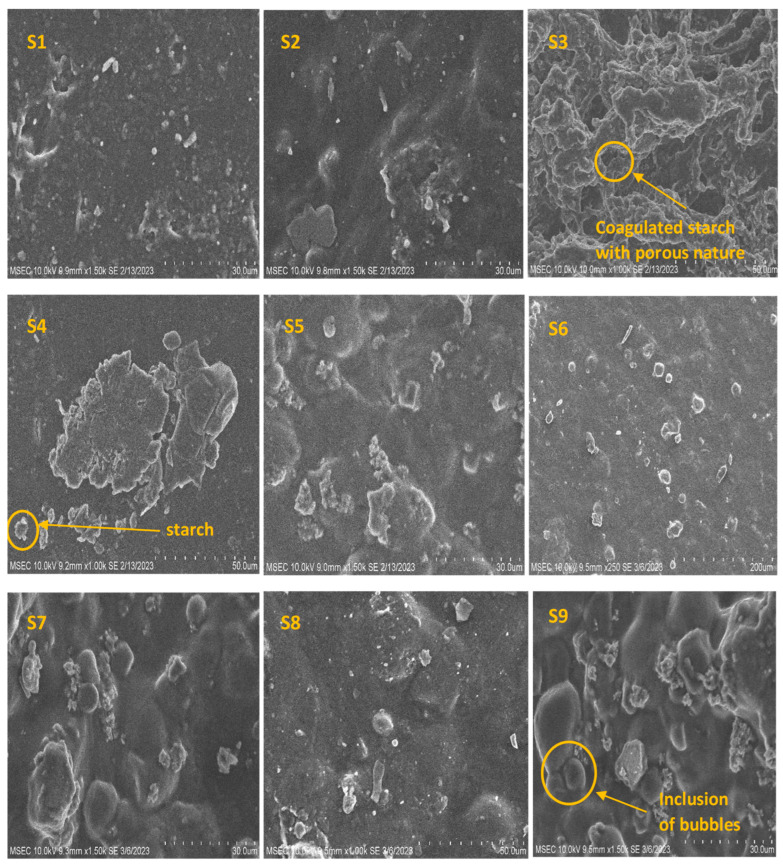
Scanning electron microscope results for various bioplastic samples (S1–30% starch, 5% glycerol, S2–20% starch, 5% glycerol, S3–18% starch, 5% glycerol, S4–26% starch, 5% glycerol, S5–30% starch, 5% sorbitol, S6–20% starch, 5% sorbitol, S7–29% starch, 5% sorbitol, S8–20% starch, 2.5% of glycerol and sorbitol, S9–30% starch, 2.5% of glycerol and sorbitol).

SEM photographs on a composite proportion of nine samples are shown in Figure 15. On analyzing these samples through SEM, it is inferred that all the samples that are produced have been well-coagulated, thereby eliminating the risks of inducing fine holes, cracks, and disintegrated particles. The composite starch (Figure 15, samples 1–9) contents are dispersed well, with plasticizers showing that a proper homogenous distribution would increase its surface integrity and smoothness. Plasticizers like glycerol and sorbitol provide enough bonding attraction on the fine coarseness of starch, unlike sorbitol, using glycerol used to have more permeability; moreover, this enables the sample to accumulate more capacity and space than its original size on exposed to the environment.

A percentage increase (1–2 g) in addition to calcium carbonate (Figure 15, Sample 3, Sample 4, Sample 7, Sample 9) from the overall percentage of components (20–30 g) increases ductility on the sample and shows reduced thickness and leaves more suspended particles. Besides the contrast towards thickness, the sorbitol-prepared sample (Figure 15, Sample 5, Sample 6, Sample 7) eliminates water inclusion because of having better bonding on the composite material, which prevents permeability. Similar types of morphology have also been discovered in earlier investigations [28]. Abnormal bulges on the samples (Figure 15, Sample 5, Sample 7, Sample 9) clearly shows that adequate mixing at continuous interval enables the formation of microbubbles onto the samples, which reflects in an uneven suspension of starch particles.

### 4.8. Fourier Transmission Infrared Spectroscopy (FT–IR)

**Figure 16:**
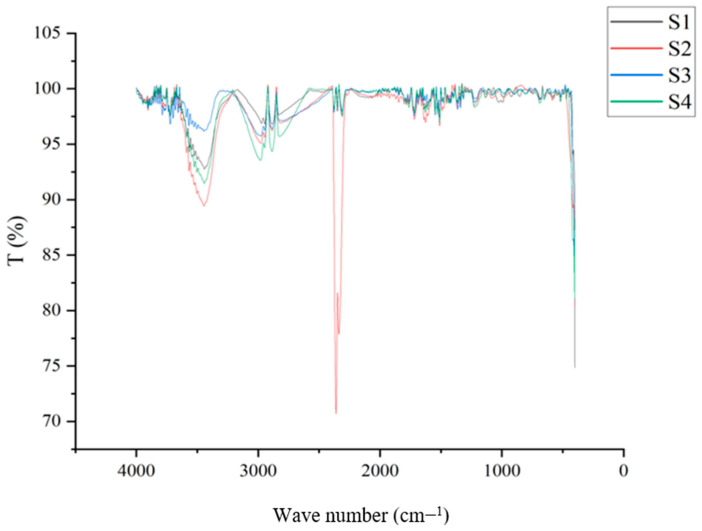
FT−IR results on samples plasticized using glycerol.

**Figure 17:**
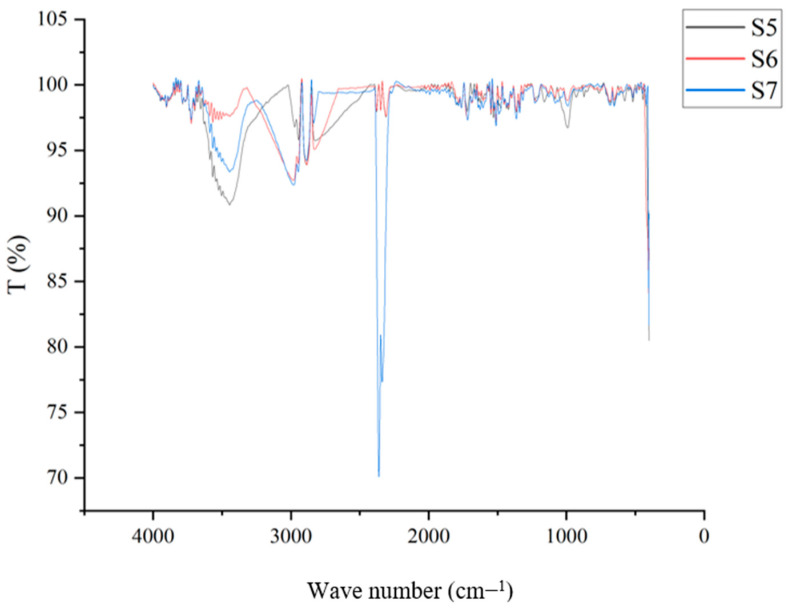
FT−IR results on samples plasticized using sorbitol.

**Figure 18:**
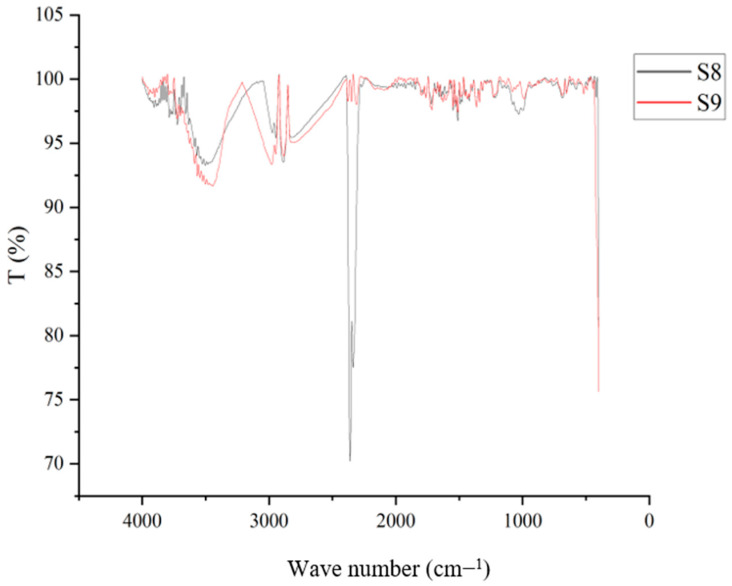
FT−IR results on samples plasticized using glycerol and sorbitol.

Based on the substances given, this experiment demonstrates how several functional groups can exist. Based on the plasticizers added to the samples, such as glycerol and sorbitol, as well as their combined nature, the graphical depiction has been categorized as shown in Figure 16, Figure 17 and Figure 18. Samples 1, 2, 3, and 4 were plasticized using glycerol, and when compared, the distinctive peaks between 3250 and 3550 cm^−1^ reflect the O-H stretching of the alcohol group. Similar peaks have also been observed in samples of starch-based bioplastic in earlier studies [68].

These peaks show that the samples include glycerol, which has a hydroxyl group. C-O-H groups can be seen at the level where between 1624 cm^−1^ and 1759 cm^−1^ pass from one to the other. The peaks between 2883 cm^−1^ and 3000 cm^−1^ indicate the presence of the C-H stretching group. The inclusion of starch has led to the presence of these groups. These would resemble the samples that had been plasticized with glycerol.

The existence of the C=O=H group is indicated by the similar characteristic peaks over Sample 2, Sample 7, and Sample 8 between 2350 cm^−1^ and 2500 cm^−1^. This could be brought on by the samples’ content of rice starch. The comparison of the graph’s fingerprint area matches the distinctive qualities of the various plasticizers employed in the samples. These findings would enable us to categorize the various functional groups based on the components of the bioplastic samples.

### 4.9. Thermal Analysis

**Figure 19:**
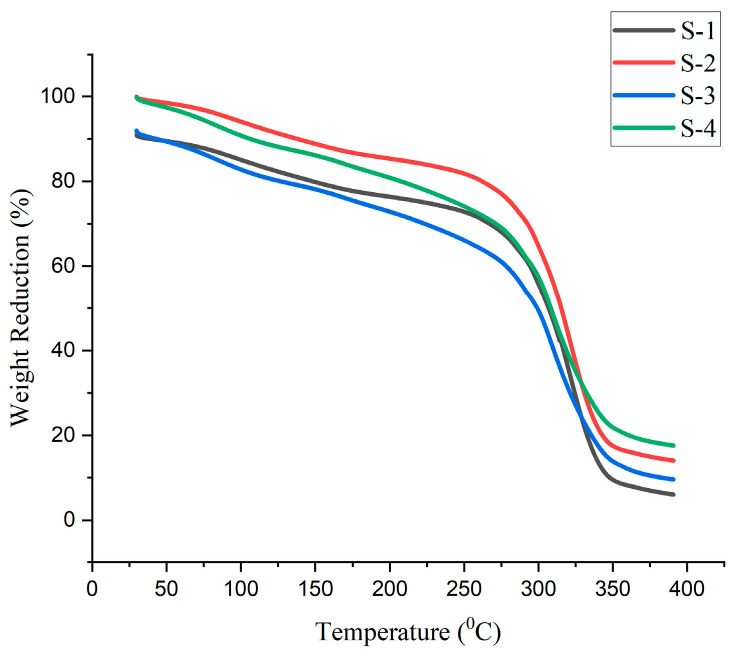
TGA curves for samples prepared using glycerol.

**Figure 20:**
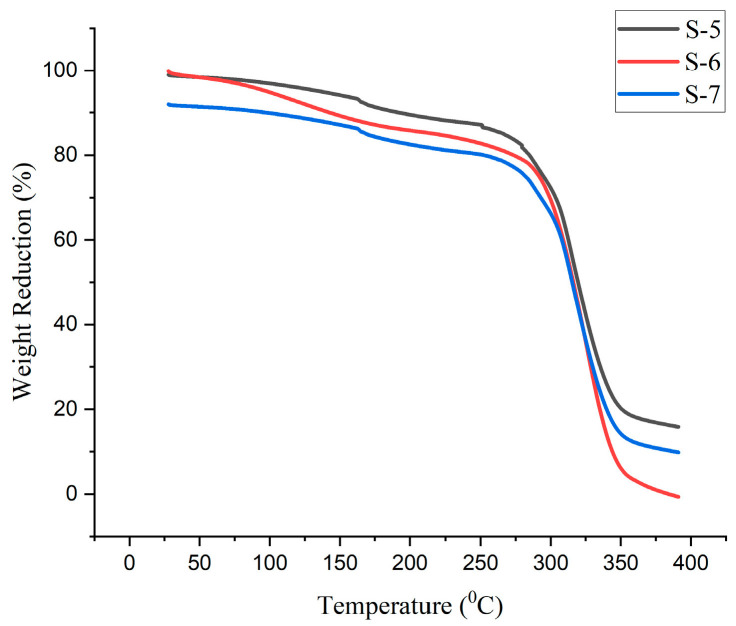
TGA curves for samples prepared using sorbitol.

**Figure 21:**
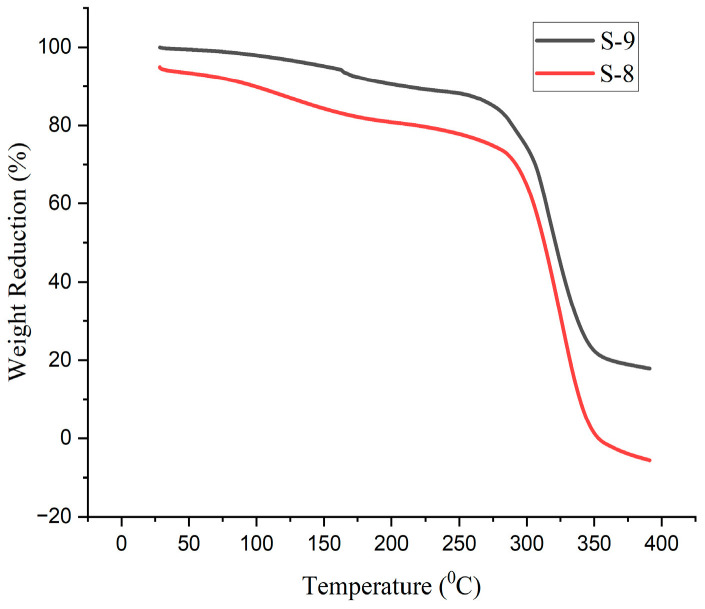
TGA curves samples prepared using glycerol and sorbitol.

The degradation of the bioplastic samples happened in three stages, at 130 °C, 290 °C, and 390 °C, as indicated by the graphs of the thermal analysis of the various compositions of the generated bioplastic samples according to Figure 19, Figure 20 and Figure 21. The plasticizers (glycerol and sorbitol) start to evaporate at 220 °C and are completely evaporated at 290 °C. The entire decomposition of the bioplastics occurred between 220 °C and 390 °C. It is observed that the evaporation of moisture of the bioplastics made the first stage at 130 °C.

This is due to the evaporation of the water. The acquired breakdown graphs revealed that 50% of weight reduction occurs at 315 °C for the glycerol-based Sample 2. The graph shows a progressive decline in weight between 220 °C and 350 °C. The temperature of 308 °C causes a 50% weight drop for the glycerol with CaCO_3_ Sample 4. For sorbitol-based samples (Sample 6), a weight decreases of 50% occurs at 316 °C. According to the research, weight loss occurs in three stages, which are more comparable to the present study [27].

The obtained results showed that 2–70% of weight reduction occurred in 45–332 °C. In total, 50% of weight reduction occurs at the temperature of 321 °C for the combination of glycerol and sorbitol-based samples (Sample 9). The decomposition temperature of the bioplastics is nearly 390 °C. From this, it could be concluded that the prepared bioplastics samples are suitable for temperatures below 390 °C.

## 5. Conclusions

Composite samples are prepared using two different starch concentration ratios (30 g, 40 g) in order to consolidate the samples in accordance with the test results. The coagulated compounds samples possess adequate mechanical properties like withstanding the tensile strength at a desired level and the capability of the samples undergoing 400 °C on thermogravimetric analysis. The following conclusions were drawn from the results.

- The scanning electron microscope image specifications show the compound binding nature and pores permeability for water inclusion. Though the characterizations of these samples are explicit to high and low of varying nature based on aggregating all these values of their results, S4 shows an acceptable proportion as in the best sample, ensuring the optimal signs for product development;
- Functional groups and potential chemical changes brought on by the addition of plasticizers and fillers were identified using the FTIR analysis. All of the bioplastic samples that were analyzed showed the characteristic peaks between 2925 and 3011 cm^−1^, indicating = C-H stretching, which is caused by the presence of starch;
- Maximum tensile strength of 13.612 MPa was achieved by Sample 6 with sorbitol bioplastic due to molecular interactions and hydrogen bonds. When calcium carbonate was added, the tensile strength decreased. Furthermore, the samples’ ability to resist tensile stress decreased with increasing starch content. In addition, the glycerol-based samples were much more flexible than those containing sorbitol;
- The corn concentration and glycerol help in the easy binding of other compounds and enable strength. Some ratios need to be at a level that is predictive of lower values, even while the sample needs better findings to be used and implemented. Examples of such constraints are a lower degradation rate and a greater water absorptivity level.

Commercialized packaging will be impacted by the development of bio-based plastic applications as substitutes for food packaging. By doing so, it would be clear that these results would have an impact on large-scale manufacturing. The effects of additional plasticizers on the mechanical properties of the created bioplastics may be investigated in further studies. Finding effective fabrication techniques is a potential additional area of attention for this work.

## Figures and Tables

**Table 1:** Composition of the bioplastics.

Sample No.	Corn Starch (g)	Rice Starch (g)	Tapioca Starch (g)	Glycerol (mL)	Sorbitol (mL)	Calcium Carbonate (g)
Sample 1	16	2	12	5	0	0
Sample 2	8	4	8	5	0	0
Sample 3	10	4	4	5	0	2
Sample 4	16	2	8	5	0	4
Sample 5	16	6	8	0	5	0
Sample 6	14	2	4	0	5	0
Sample 7	17	4	8	0	5	1
Sample 8	12	4	4	2.5	2.5	0
Sample 9	16	4	8	2.5	2.5	2

**Table 2:** Normalizing sequence.

Samples	Solubility in Water	Solubility in Alcohol	Absorption of Water	Moisture Content	Tensile Strength	Biodegradability
Sample 1	0.000	0.056	0.000	0.000	0.159	0.000
Sample 2	0.361	0.000	0.876	0.306	0.512	1.000
Sample 3	0.656	0.851	0.723	0.455	0.215	0.986
Sample 4	0.484	0.125	0.746	0.156	0.360	0.736
Sample 5	0.818	0.469	0.938	0.414	0.132	0.047
Sample 6	0.401	0.447	0.166	0.651	1.000	0.716
Sample 7	0.942	0.765	1.000	0.489	0.000	0.303
Sample 8	0.330	0.804	0.008	0.781	0.215	0.541
Sample 9	1.000	1.000	0.661	0.609	0.122	0.104

**Table 3:** Deviation sequence.

Samples	Solubility in Water	Solubility in Alcohol	Absorption of Water	Moisture Content	Tensile Strength	Biodegradability
Sample 1	1.000	0.944	1.000	1.000	0.841	1.000
Sample 2	0.639	1.000	0.124	0.694	0.488	0.000
Sample 3	0.344	0.149	0.277	0.545	0.785	0.014
Sample 4	0.516	0.875	0.254	0.844	0.640	0.264
Sample 5	0.182	0.531	0.062	0.586	0.868	0.953
Sample 6	0.599	0.553	0.834	0.349	0.000	0.284
Sample 7	0.058	0.235	0.000	0.511	1.000	0.697
Sample 8	0.670	0.196	0.992	0.219	0.785	0.459
Sample 9	0.000	0.000	0.339	0.391	0.878	0.896

**Table 4:** Grey Relational Coefficient.

Samples	Solubility in Water	Solubility in Alcohol	Absorption of Water	Moisture Content	Tensile Strength	Biodegradability
Sample 1	1.000	0.899	1.000	1.000	0.759	1.000
Sample 2	0.580	1.000	0.363	0.620	0.494	0.333
Sample 3	0.433	0.370	0.409	0.524	0.700	0.336
Sample 4	0.508	0.800	0.401	0.762	0.581	0.405
Sample 5	0.379	0.516	0.348	0.547	0.791	0.913
Sample 6	0.555	0.528	0.750	0.434	0.333	0.411
Sample 7	0.347	0.395	0.333	0.506	1.000	0.623
Sample 8	0.602	0.383	0.985	0.390	0.699	0.480
Sample 9	0.333	0.333	0.431	0.451	0.804	0.827

**Table 5:** Response for Grey Relational Grade.

Level	Starch	Glycerol	Sorbitol	Filler
1	0.6567	0.5792	0.7225	0.6851
2	0.5535	0.498	0.5261	0.5338
3	0.5513	0.6844	0.5653	0.5427
Delta	0.1054	0.1864	0.1965	0.1513
Rank	4	2	1	3

**Table 6:** Grey Relational Grade.

Samples	Grade
Sample 1	0.943
Sample 2	0.565
Sample 3	0.462
Sample 4	0.576
Sample 5	0.582
Sample 6	0.502
Sample 7	0.534
Sample 8	0.590
Sample 9	0.530

## Data Availability

The data presented in this study are available on request from the corresponding author.
